# Microbial dysbiosis in the gut drives systemic autoimmune diseases

**DOI:** 10.3389/fimmu.2022.906258

**Published:** 2022-10-20

**Authors:** Walaa K. Mousa, Fadia Chehadeh, Shannon Husband

**Affiliations:** ^1^ Biology Department, Whitman College, Walla Walla, WA, United States; ^2^ College of Pharmacy, Al Ain University, Abu Dhabi, United Arab Emirates; ^3^ College of Pharmacy, Mansoura University, Mansoura, Egypt

**Keywords:** microbiome, autoimmune diseases, dysbiosis, T1D, MS, SLE, arthrities

## Abstract

Trillions of microbes survive and thrive inside the human body. These tiny creatures are crucial to the development and maturation of our immune system and to maintain gut immune homeostasis. Microbial dysbiosis is the main driver of local inflammatory and autoimmune diseases such as colitis and inflammatory bowel diseases. Dysbiosis in the gut can also drive systemic autoimmune diseases such as type 1 diabetes, rheumatic arthritis, and multiple sclerosis. Gut microbes directly interact with the immune system by multiple mechanisms including modulation of the host microRNAs affecting gene expression at the post-transcriptional level or production of microbial metabolites that interact with cellular receptors such as TLRs and GPCRs. This interaction modulates crucial immune functions such as differentiation of lymphocytes, production of interleukins, or controlling the leakage of inflammatory molecules from the gut to the systemic circulation. In this review, we compile and analyze data to gain insights into the underpinning mechanisms mediating systemic autoimmune diseases. Understanding how gut microbes can trigger or protect from systemic autoimmune diseases is crucial to (1) tackle these diseases through diet or lifestyle modification, (2) develop new microbiome-based therapeutics such as prebiotics or probiotics, (3) identify diagnostic biomarkers to predict disease risk, and (4) observe and intervene with microbial population change with the flare-up of autoimmune responses. Considering the microbiome signature as a crucial player in systemic autoimmune diseases might hold a promise to turn these untreatable diseases into manageable or preventable ones.

## Introduction

The gastrointestinal tract is home to trillions of diverse microbial species including bacteria, viruses, and fungi (1–3). To colonize the human body, these microbes interact with the immune system to develop tolerance and maintain gut homeostasis. Gut microbes, or their secreted metabolites, directly interact with gut-associated lymphoid tissues (GALTs), which include Peyer’s patches, mesenteric lymph nodes, intraepithelial lymphocytes, lamina propria, and isolated lymphoid follicles (4, 5). This interaction is mediated through Toll-like receptors (TLRs), leading to induction of immune cell differentiations, setting the balance between helper T cells and regulatory T cells (6). Moreover, gut microbes modulate genes involved in maintaining the mucosal barrier function such as those involved in the synthesis of the tight junctions (6, 7) and formation of mucin barrier (8, 9).

Gut microbes could also modulate host genes through microRNAs (miRNAs), short non-coding RNA sequences that silence gene expression (10, 11). Host-derived miRNAs serve an essential function to control microbial population abundance by directly affecting microbial gene expression and mucosal colonization (12–15). Microbes, or their secreted metabolites, modulate miRNAs affecting host genes. The affected host genes include those linked to differentiation of T cells, production of interleukins, proliferation of intestinal epithelial cells, gut permeability, and autophagy process (16–21). Selected examples of miRNAs mediating host–microbe interactions leading to or preventing inflammation are noted and summarized (21–34) (**Table 1**).

**Table 1:** The role of selected miRNAs in shaping the microbiome structure and mediating inflammatory and autoimmune diseases.

Unique miR	Molecular mechanism	Outcome	Reference
miR-515-5p and miR-1226-5p	Upregulate growth-related genes in *F. nucleatum* and *E. coli*	Drives inflammatory bowel diseases and colorectal cancer	(21, 22)
miR-21	Control of gut microbes	Drives colitis	(23)
miR-193a-3p.	Interferes with the ability of the intestinal cells to absorb L-Ala-γ-D-Glu-meso-DAP, a proinflammatory tripeptide	Reduces inflammation	(24, 25)
miR-375-3p	Promotes proliferation of the intestinal epidermal cells		(19)
miR-21-5p	Increases permeability of the intestinal epidermal cells	Drives inflammation	(26)
miR-106b	Affects expression of p21 gene, which mediates the anti-inflammatory effect of microbial short-chain fatty acids	Control of host genes	(20)
miR-150 and miR-143	Upregulated by *Lactobacillus salivarius* and *L. fermentum*	Reduces inflammation in colitis mouse model	(27)
miR-18a and miR-4802	Downregulated by *Fusobacterium nucleatum*	Interference in autophagy pathways Increases resistance to chemotherapeutics	(21)
miR-21	Modulated by *F. nucleatum*	Increases level of prostaglandin E2 and IL-10 Inhibits anti-tumor T-cell response leading to progression of cancer	(28)
miR-20a-5p	Overexpressed by some strains of *E. coli*	Enhances expression of some growth factors leading to colorectal cancer	(29)
miR-10a	Downregulated by commensal microbes	Targeting IL-12/IL-23p40 contributing to the immune homeostasis	(17)
miR-let 7f	Downregulated by *M. tuberculosis*	Decrease in the production of tumor necrosis factor and IL-1Beta suppressing the immune system	(30)
miR-141 and miR-200a	Inducers of Th17 differentiations and repressors for Treg cells	Leading to progression of MS	(31)
miR-155 inflammation ^38^	Regulates Th17/Treg balance through toll-like receptors (TLRs), the sensor of gut innate immunity	Over-expression enhances Th17 immunogenic function and suppresses Treg cells	(32, 33)
miR-18b, miR-363-3p, and miR-106a	These miRs suppress differentiation of Th17 and subsequent inflammation	Decreases the production of proinflammatory interleukins IL17 resulting in anti-inflammatory effect	
miR-1, miR-27a and b, miR-30c, and miR-141	Predicted to induce Th17 differentiation	Drives inflammation	(34)
miR-20a, miR-20b, miR-21, miR-93, miR-106a, and miR-152	Predicted to suppress Th17 differentiation	Suppress inflammation	(34)

**Figure 1:**
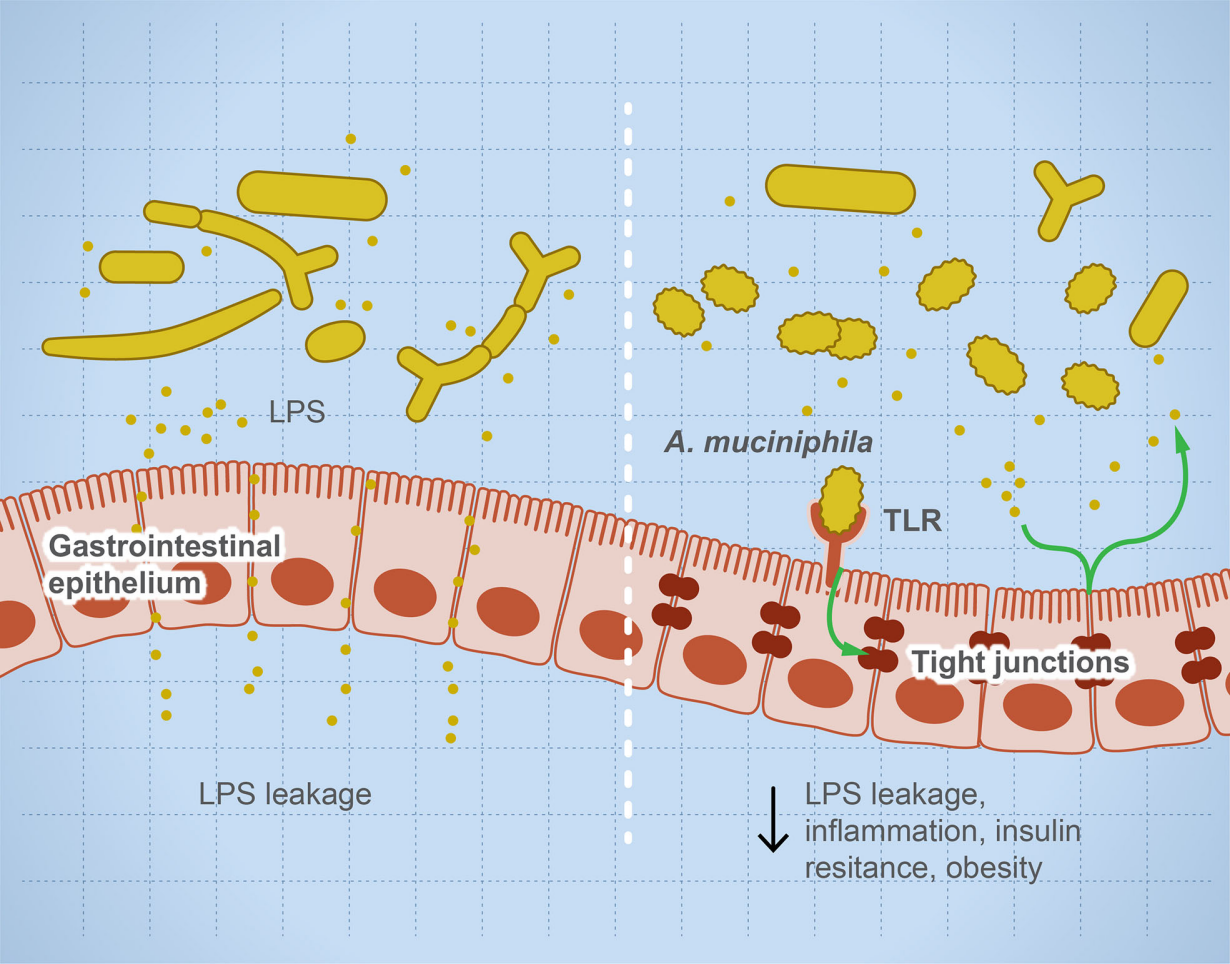
Microbial dysbiosis drives systemic inflammation by targeting the mucosal barrier. The illustration represents that, during microbial dysbiosis, the gut barrier is leaky, which results in diffusion of microbial metabolites such as lipopolysaccharides into the circulation, causing systemic inflammation. However, healthy microbiome has a balanced microbial composition including potential anti-inflammatory microbes such as *Akkermansia muciniphilia* and *Faecalibacterium prausnitzii*. These microbes or their metabolites activate TLRs leading to overexpression of tight junction proteins and prevent gut leakage. This leakage is thought to drive systemic inflammatory and autoimmune diseases such as insulin resistance and obesity.

Microbial dysbiosis disturbs the immune function leading to inflammation and sensitization of the immune system and causing autoimmune diseases (35, 36). Many factors influence microbial dysbiosis such as diet, stress, drugs, diseases, age, and lifestyle. The imbalance in helper T cells/regulatory T cells drives autoimmune diseases such as colitis and multiple sclerosis (MS) (18, 33). Leakage of metabolites such as lipopolysaccharides sensitizes the immune system, leading to a higher production of pro-inflammatory interleukins, and degradation of mucin resulting in irritation of the gut lining and microbial invasion (37). **Figure 1** illustrates the role of some microbial taxa in maintaining gut barrier function and how microbial dysbiosis results in a leaky gut. Each microbe prevents or drives inflammation by a unique mechanism. For example, *Faecalibacterium prausnitzii* prevents inflammation by inducing Treg differentiation, leading to the subsequent higher production of IL-10 (an anti-inflammatory interleukin) (38, 39). In contrast, *Fusobacterium nucleatum* drives inflammation by inhibiting cytotoxic T cells and modulation of miRNAs, leading to suppression of autophagy (28). Several examples of individual microbes that modulate host immune response to prevent or drive inflammation and autoimmune reaction are noted (40–72) and summarized (**Table 2**).

**Table 2:** Unique gut microbes as drivers or inhibitors of autoimmune diseases.

Microbe	Role of the microbiota in modulating inflammation	References
*Lactobacillus casei*	Synergizes with poly (I:C), a TLR3 ligand, to promote Th1 response leading to selective enrichment in IL-12p70 production.	(45)
*Faecalibacterium prasuntzii*	Silences NF-κB gene expression through inhibition of HDACS, which results in hyperacetylation of NF-κB-encoding gene preventing its expression. *F. prausuntzii* exerts anti-inflammatory activity by inducing Treg cells and through butyrate production.	(38, 46)
*Bacteroides thetaiotaomicron*	Degrades carbohydrate to produce butyrate.	(39)
*Roseburia* genera	Decreases inflammation through production of butyrate.	(47, 48)
*Lactobacillus salivarius* and *L. fermentum*	Anti-inflammatory activity by enhancing expression of miR-150 and miR-143 in a mouse model of colitis. Restoration of the gut barrier function.	(27, 49)
*Desulfovibrio piger*	Associated with higher level of plasma 1-arachidonoyl-GPC levels, a metabolite known to negatively affect CD4+ CXCR3+ and CD8+ CXCR3+ T cells^50^, preventing further progression of autoimmunity.	(50)
*Bacillus cereus*	Associated with delayed onset of T1D in NOD mice.	(51–53)
*Akkermansia* sp.	Increases butyrate production and can protect against pancreatic autoimmunity.	(54)
*Prevotella histicola*	Prevents arthritis in mice. *P. histicola* regulates dendritic cells (CD103+) resulting in generation of Treg cells, which suppresses TH17, decreasing proinflammatory interleukins and increasing anti-inflammatory interleukins such as IL-10.	(55)
*Bifidobacterium bifidum*	Inhibits excessive stimulation of CD4+ lymphocytes.	(56)
*F. nucleatum*	Inhibits anti-tumor T-cell response, leading to progression of cancer through a modulatory effect on miR-21, which increases level of prostaglandin E2 and IL-10, although the exact mechanism is not clear. Stimulates expression of NF-κB gene through miRNA, which results in inflammation.	(21, 22, 28)
*M. tuberculosis*	Decreases miR-let 7f in infected microphages, leading to a decrease in the production of tumor necrosis factor (TNF) and IL-1Beta, which suppresses the immune system by affecting NF-κB inflammatory response.	(30)
*Solobacterium moorei*	Diagnostic pathobiont in inflammatory bowel diseases.	(57)
Adherent-invasive *Escherichia coli* (AIEC)	Induces inflammation by irritating the gut lining. AIEC also produces propionates, an SCFA stimulating the production of IL-1β, a component of the inflammasome that increases the production of IL-18.	(20)
Cytophaga-flavobacter-bacteroides (CFB)	Affects TH17/Treg balance, which is crucial for immune homeostasis leading to autoimmune diseases. CFB induces Th17 differentiations, and its absence is associated with induction of Treg cells in the lamina propria.	(58)
*Bacteriodes dorei*	Produces LPS that induces the innate immune response.	(59)
*Provetella* species	Breakdown of mucin and contribute to intestinal inflammation.	(60, 61)
Segmented filamentous bacteria	Produces serum amyloid protein A, which increases the production of Th1 and Th17 that migrate systemically and contribute to systemic autoimmune diseases.	(62)
*Provetella copri*	Stimulates differentiation of TH17, leading to excessive productions of proinflammatory interleukins such as IL-23 and IL-1 and recruitment of neutrophil.	(63–67)
*Prevotella intestinalis*	Implicated in colitis through reduction in short-chain fatty acids and the anti-inflammatory IL-18 in mice.	(68)
*Enterococcus gallinarum*	Translocate from the gut to the liver, resulting in overproduction of autoimmune antibodies, inflammation, and mortality in genetically susceptible mice.	(69)
*Eggerthella lenta* and *Akkermansia muciniphila*	Increases in MS patients. MS patients show increase in anti-*A muciniphila* immunoglobulin G.	(70–72)
*Akkermansia muciniphila*	Interacts with spore-forming bacteria to escalate the inflammation leading to MS through direct effect on T lymphocytes.	(73)
*Lactobacillus bifidus*	Activates autoimmune response in IL-1 receptor antagonist-knockout mice.	(74, 75)
*Ruminococcus gnavus*	Enriched in genes for the production of proinflammatory polysaccharides and lower potential in fiber-degrading enzymes.	(76)

## Microbial dysbiosis drives systemic autoimmune diseases

Microbial dysbiosis is strongly linked to local inflammation and autoimmune diseases in the gut such as Crohn’s and inflammatory bowel diseases. However, much less is known about the link between dysbiosis in the gut and systemic autoimmune diseases. Evidence suggests that gut microbes exert some control over the systemic immune response, particularly innate immunity. To induce immune tolerance to commensal microbes, the antigens of the gut microbes are sampled by dendritic cells (DCs) and presented to T cells in the pancreatic lymph nodes (PLNs) (77, 78). Sometimes, T cells activated by microbial antigen fragments spread and trigger a systemic immune response. Additionally, some microbial metabolites might leak from the gut barrier to other tissues or organs. Short-chain fatty acids (SCFAs) suppress neutrophil function *via* binding to GPCRs (79) while microbial peptidoglycans stimulate neutrophil function *via* Nod1 (40). Here, we discuss links between microbial dysbiosis and some systemic autoimmune diseases including type 1 diabetes (T1D), Multiple Sclerosis (MS), rheumatoid arthritis (RA), and systemic lupus erythematosus (SLE).

### The role of microbial dysbiosis in triggering type 1 diabetes

**Figure 2:**
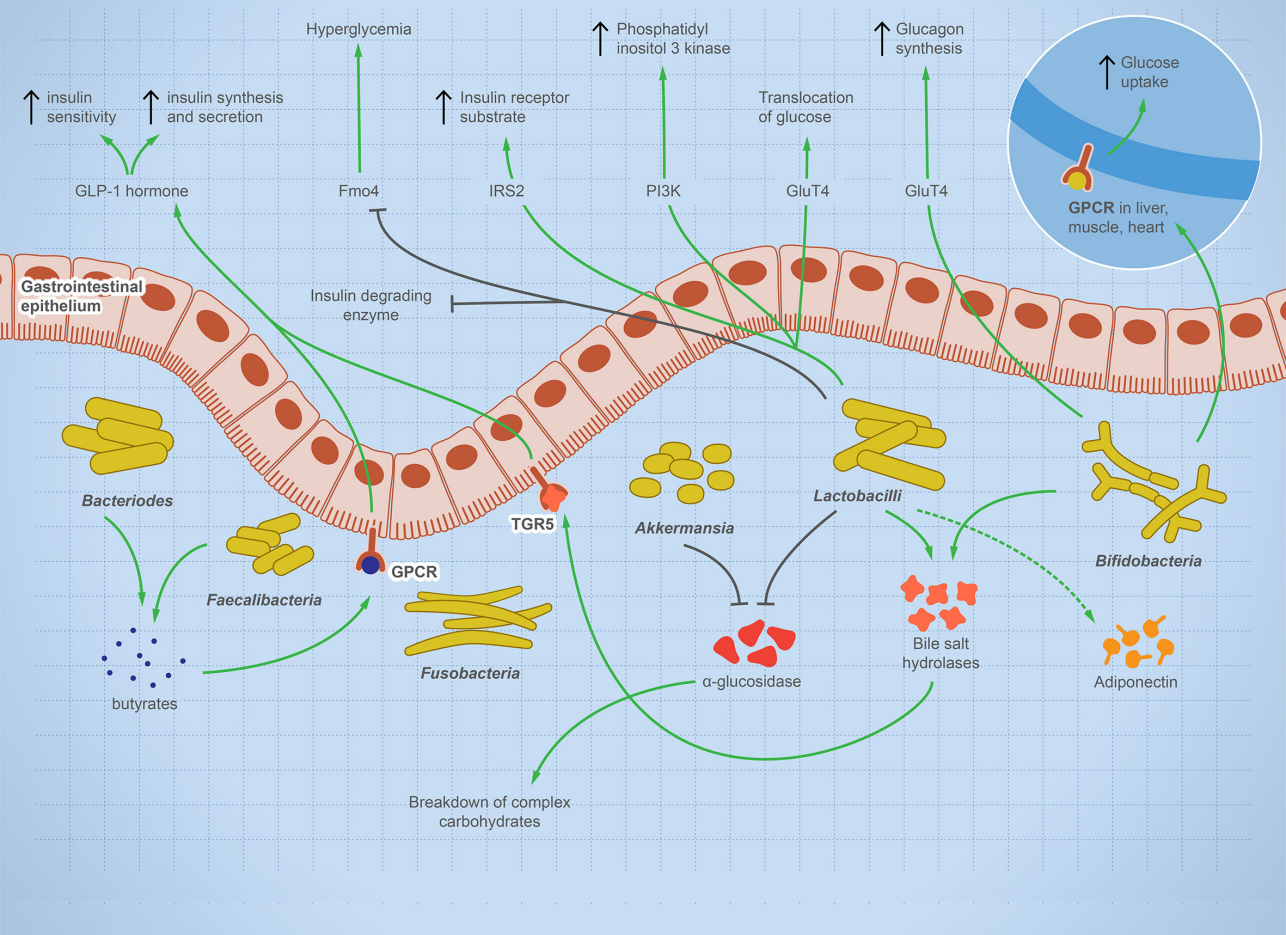
The integral role of the balanced gut microbes in maintaining glucose level and suppressing hyperglycemia. The illustration shows the fundamental role of some representative gut microbes in glucose metabolism. The onset of T1D is characterized by a change in microbial composition characterized by decrease in specific taxa such as *Bifidobacterium, Lactobacilli, Bacteroides, Faecalibacterium*, and *Akkermansia—*while other taxa such as *Fusobacterium* is enriched. *Bifidobacterium* increases glucose uptake in heart, muscle, and liver tissues and stimulates glucagon synthesis. *Lactobacilli* stimulates cellular receptors involved in translocation of glucose, production of phosphatidyl inositol-3-phosphate, production of insulin receptor substrate, and suppression of hyperglycemia. *Bacteroides* and *Faecalibacterium* produce butyrate, which binds to GPCR, activating the production of GLP-1 hormone, which is involved in insulin synthesis, secretion, and sensitivity. *Lactobacilli* and *Akkermansia* have an inhibitory effect on alpha-glucosidase enzyme, which helps in the breakdown of complex carbohydrate, raising sugar level. *Lactobacilli* and *Bifidobacterium* increase bile salt hydrolyses, leading to GLP-1 hormone stimulation. If this microbial role is disturbed, inflammation and autoimmune diseases arise.

T1D is a systemic autoimmune disease that is linked to microbial dysbiosis. Preclinical and clinical T1D is mostly associated with GIT pathogenesis, such as celiac disease or increased intestinal leakage potentially due to microbial dysbiosis (41–43). Multiple studies report significant shifts in gut microbes including bacteria, viruses, and fungi before the onset of T1D as reviewed (44). **Figure 2** illustrates the balanced microbial interaction that contributes to glucose metabolism and suppresses hyperglycemia.

A study on mice reported that cytophaga-flavobacter-bacteroidetes (CFB) affects Th17/Treg balance, which is crucial for immune homeostasis, leading to autoimmune diseases (58). CFB induces Th17 differentiation, and its absence is associated with induction of Treg cells in the lamina propria, and this effect is diminished after selective antibiotic treatment (58). Another study reported an enrichment in *Bacteriodetes dorei* with preclinical or clinical T1D. *B. dorei* produces LPS that induces the innate immune response (58). The abundance of *Desulfovibrio piger* is associated with a higher level of plasma 1-arachidonoyl-GPC, a metabolite known to negatively affect CD4+, CXCR3+, CD8+, and CXCR3+ T cells (50), preventing further progression of autoimmunity (50). A similar effect of this metabolite is reported in mice (80). *D. piger* is also known to produce hydrogen sulfide, which affects T cells and immune response (81–83).

A study reported that T1D patients show enrichment in Proteobacteria, Actinobacteria, and Bacteroidetes and lack of butyrate-producing bacteria (84). Knowing that butyrate induces mucin formation and assembly of tight junction explains the increased gut permeability in T1D patients. T1D microbiota is also deficient in *Provetella* species that break down mucin and enriched in bacteria that produce propionates and acetates known to impair neutrophil functions observed in T1D patients (60, 61). Other evidence suggests that microbial dysbiosis alters the gut immunity, resulting in excessive stimulation of inflammatory response leading to T1D even without an observable change in gut permeability. For example, a study reported that T1D patients show a high expression level of intercellular adhesion molecule-1, HLA-DR, HLA-DP, IL-4, and IL-1α-positive cells (60, 85). Another study showed that DCs are not able to induce FoxP3+Treg cell differentiation in T1D patients (86). The authors claimed that the deficiency of Treg cells in the gut decreases the ability of the immune system to tolerate and discriminate self-antigens in the pancreatic β-cells (86).

A mouse model of T1D has a reduction in IL-22, IL-17A, and IL-23A that is associated with loss of segmented filamentous bacteria (87). This alteration is reversed by treatment with anti-inflammatory drugs, suggesting that dysbiosis is linked to inflammation rather than to T1D (87). Another study shows that the immunomodulatory compound, indole-3-carbinol, binds to aryl hydrocarbon receptor (AhR) in NOD mice (88). AhR is a transcription factor that prevents T1D. Interestingly, this activation was mainly localized to the small intestine (88). Meanwhile, no alteration was observed in the differentiation of T cells in the spleen or PLNs (88). These changes were associated with a signature trans-kingdom network characterized by a reduction in ruminiclostridium, intestinimonas, and lachnospiraceae mediated by an increase in CD25 (88). A study found that prediabetes in rats is associated with enrichment in *Bifidobacterium, Lactobacillus*, and *Bacteroides* species and antibiotic treatment decreased the incidence of T1D (89, 90). Other research shows that *Lactobacillus bifidus* can activate the autoimmune response in IL-1 receptor antagonist-knockout mice (74, 75).

Mice that are deficient in MyD88, a specific adapter molecule involved in TLRs signaling pathways, are protected from T1D in the presence of a microbiome signature characterized by a richness in *Lactobacillus* genera and low Firmicutes/Bacteroidetes ratio (91). Interestingly, *Lactobacillus* shows protection against T1D while *Bacillus cereus* delayed the onset of T1D in NOD mice (51–53). Metagenomic analysis revealed that stool samples from T1D patients are enriched in genes responsible for bacterial adhesion and flagella formation that are possibly involved in triggering systemic immune response (84). T1D patients are also deficient in butyrate producers and mucin-degrading bacteria (84).

Preclinical T1D is also associated with a reduction in butyrate-producing genera such as *Roseburia* (48). When dietary fibers (DF) intake is limited, gut microbes degrade mucin to increase the supply of butyrate. Mucin degradation increases gut permeability and immunogenicity leading to T1D (92, 93). Butyrate modulates susceptibility to T1D through a variety of possible mechanisms such as upregulation of tight junction proteins (94). Butyrate enhances differentiation of Treg cells through histone H3 acetylation (95, 96) and induces apoptosis of proinflammatory T cells in the murine cell line (97). Another study shows that *Akkermansia* sp., a butyrate producer, protects against pancreatic autoimmunity (54). Butyrate promotes Th1 differentiation through induction of IFN-γ and T-bet expression by inhibition of histone deacetylase. Butyrate inhibits Th17 differentiation through suppression of Rorα, Rorγt, and IL-17 (98). The literature shows mixed results for the effect of oral administrations of butyrate on the development of T1D in humans versus experimental animals (99).

Several reports suggest that viral infections can trigger or attenuate the development of T1D or pancreatic autoimmunity, particularly infection with rotavirus, cytomegalovirus (CMV), and enterovirus, although the underpinning mechanisms are not well-understood (100–104). Several findings in experimental animals, particularly NOD mice, suggest that the viruses affect T1D development by molecular mimicry or bystander inflammation. Molecular mimicry is reported for molecules such as glutamic acid decarboxylase enzyme, tyrosine phosphatase IA-2/IAR, and heat shock protein 60 (105, 106). For example, Coxsackievirus B4 (CVB4) triggers T1D by mimicry to host-related molecules such as beta-cell glutamic acid decarboxylase enzyme 65 (105, 106). Interestingly, the adequate response of pancreatic B cells to interferon-gamma reduced CVB4-induced T1D (107). Another hypothesis is that the virome induces pancreatic bystander inflammation, which involves the inability of CD8+ cytotoxic T lymphocytes to recognize self-antigens of the pancreatic beta cells. Interestingly, there are some reports of viral infections linked to less incidence of T1D such as CXADR rs6517774, Mastadeno-virus C, and Norovirus-4 (103, 108). In the NOD mouse model, infection with mouse Norovirus-4 (MNV4) is associated with enrichment in the alpha diversity of gut bacteria and expansion of Treg cells (108). To examine if the effect of MNV4 on immunity is due to indirect modulation of the gut microbes, the authors repeated the experiment on germ-free mice. Interestingly, germ-free mice infected with MNV4 still show modulation of the immune response including an increase in Treg and alteration in cellular and secreted components of the immune system such as B cells, T cells, macrophages, and cytokine biomarkers (108).

Dysbiosis in the virome is also associated with several autoimmune diseases including T1D (109, 110). The virome is the collection of endogenous retroviruses, eukaryotic viruses, and bacteriophages inhabiting the gut microbiota (110, 111). Most of the gut viruses are sourced from lysogens; the latter is defined as bacteria containing dormant phages (prophages) inserted in the bacterial genomes. These phages are released from the lysogens upon receiving particular signals, including a change in diet, and further affect microbiome structure and susceptibility to autoimmune diseases (112). Prophages might encode immune-modulatory molecules that directly affect the immune system response. For example, the novel prophage ΦHKU.vir encodes toxin with superantigens that enable colonization of *S. pyogenes* by inducing nonspecific differentiation of T cells (113). The virome and host–disease associations are much less studied compared to the gut microbiome. Moreover, the literature shows mixed results and a non-confirmed link between virome shift and T1D. The main challenge in understanding virome interaction is that it directly affects bacterial population dynamics and diversity, increasing confounding factors and making it hard to draw a confirmed conclusion (111, 114). Phage infection of gnotobiotic mice, inoculated with defined human microbes, resulted in direct reduction of the susceptible species and indirect effect on the other species likely through interspecies interactions (114). Another study reported an association between the initial abundance of amyloid-producing *E. coli* and *E. coli* bacteriophage/*E. coli* ratio, which results in depletion of *E. coli*, in the development of T1D (115).

In summary, many studies investigate the role of microbial dysbiosis in triggering T1D through a variety of mechanisms such as modulation of Th17/Treg balance, variation in interleukin production, and change in gut permeability. These effects are mediated by some microbial metabolites such as butyrate, LPS, and arachidonoyl-GPC. Activation of latent viral infections can also drive microbial dysbiosis leading to T1D. However, less is known about microbial dysbiosis in late-onset T1D and if diet management can modulate disease severity or progression (116). A study reported an enrichment in *Veillonella* and *Clostridium* genera coupled with a reduction in *Bifidobacterium*, *Lactobacillus*, and *Prevotella* in the pediatric T1D compared to healthy children (117). Another study reported similar results in adults, particularly the *Bifidobacterium* signature (118). However, all studies investigating the association between late-onset T1D and microbiota shift lack directionality as T1D is thought to drive microbiota change as the diseases progress.

### The role of microbial dysbiosis in triggering multiple sclerosis

**Figure 3:**
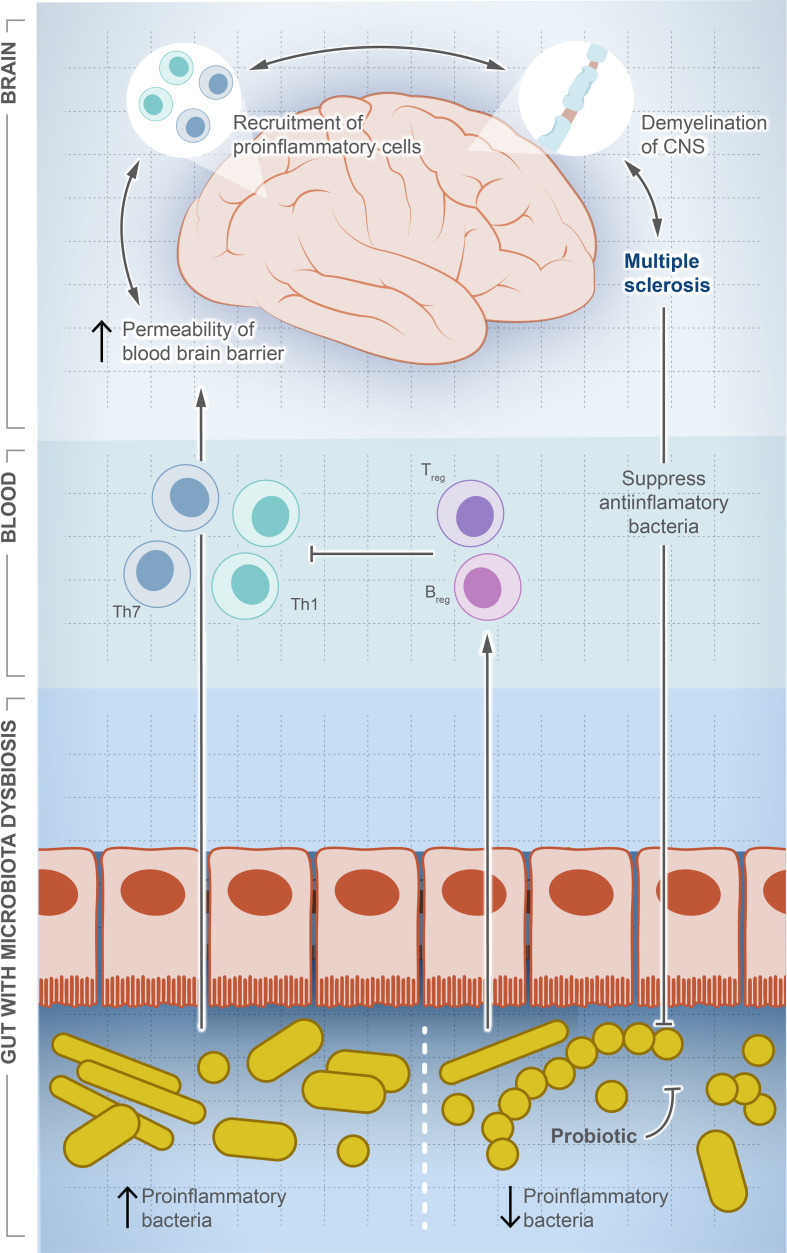
Microbial dysbiosis is a potential factor driving multiple sclerosis. In this illustration, we show how the change in population dynamic of gut microbes suppresses or drives multiple sclerosis. The increase in proinflammatory bacteria induces differentiation of Th1/Th17, which travel systemically to the brain and recruit more proinflammatory cells producing inflammatory cytokines. In contrast, the decrease in proinflammatory bacteria induces differentiation of Treg cells and production of anti-inflammatory cytokines, which balance or counteract Th1/Th17. Interestingly, once MS is developed, a significant decrease in anti-inflammatory community is observed, but the exact signaling mechanism is unknown.

MS is a chronic autoimmune disease affecting 2.8 million people around the world (119). MS causes demyelination of neurons, leading to neuroaxonal degeneration in the brain and spinal cord, resulting in an unpredictable outcome that can result in a permanent disability (120, 121). Although there is no cure for MS, some interventions can improve the quality of life and reduce complications such as anti-CD20 monoclonal antibodies, which destroy circulating memory B cells and subsequently weaken the immune system and increase the risk of infection (122). The underlying causes are largely unknown and thought to be linked to genetic factors and/or viral infection (123, 124). **Figure 3** illustrates the potential role of gut dysbiosis in MS, although the directionality of this interaction is not clear. Gut microbes are definitely altered in MS patients, and this alteration is associated with the varied severity of the disease (125–128). The microbiome signature in MS patients is characterized by the lower abundance of *F. prausnitzii*, *Prevotella*, and *Bacteroides*, and a higher abundance of *Akkermansia muciniphila* (126, 128). Pediatric MS patients show a signature reduction in SCFA-producing *Ruminococcaceae* compared to healthy children (129). However, the directionality of this association is not clear. Moreover, MS-modifying drugs can alter the microbiota composition. Administration of dimethyl fumarate and glatiramer acetate resulted in a significant reduction in Lachnospiraceae and Veillonellaceae (130).

Mechanistically, some evidence suggests the role of miRNAs such as miR-141, miR-200a, and miR-155 in driving MS by shifting Th17/Treg balance towards the Th17 side, promoting production of the proinflammatory mediator IL17 (31) (**Table 1**). These miRNAs are implicated in interfering with repressor proteins that regulate Th17 differentiation (31-33). As microbes regulate miRNAs, there is a hope that probiotics, antibiotics, or specialized microbial metabolites might suppress Th17 production or induce Treg differentiation by checking MS progression. Another possible mechanism of how microbial dysbiosis drives MS might be through molecular mimicry. Studies on neuromyelitis optica, a degenerative autoimmune disease that results in inflammation, demyelination, and nerve necrosis, suggest a possible molecular mimicry between brain and microbial antigens (131). Neuromyelitis optica is characterized by the presence of IgG1 autoantibodies that attack aquaporin 4 (AQP4), a predominant water channel in the CNS (132). This attack results in deposition of immunoglobulin complements causing demyelination and tissue damage (133, 134). The mechanism involves a molecular mimicry between AQP4 and ABC transporter permease in the gut microbe, *Clostridium perfringens* (131).

Transfer of microbiota from MS patients to mice elicits an immune response and inflammation (6). Opposing this finding, another research shows that the transfer of microbiota from a mouse model of MS to another healthy mouse resulted in disease protection thanks to miR-30d (135). The unexpected effects of this miR could be attributed to its stimulatory effect on the growth of *Akkermansia muciniphila*, which exhibits an anti-inflammatory role (135). Another possible mechanism for MS protection might be the induction of anti-inflammatory Treg cells (135). Other cohort studies show enrichment in *Eggerthella lenta* and *Akkermansia muciniphila* in MS patients (70, 71). MS patients show an increase in anti-*A muciniphila* immunoglobulin G while no difference in IgG is noted for other gut microbiota such as *Bacteroides fragilis*, *Fusobacterium*, and *Acinetobacter baumannii* (72). Other studies suggest that certain gut microbiota such as *A. muciniphila* interacts with spore-forming bacteria to escalate the inflammation leading to MS through a direct effect on T lymphocytes (73). Breakthrough research reported that some gut microbiome taxa enriched in MS patients directly interact with IgA-producing cells at the gut lining; the latter translocate to the brain cells and locally produce immunoglobulin A (IgA), which mediates severe inflammation. MS patients show a signature decrease in *Prevotella* genera compared to healthy control. Although this association lacks directionality, it varies depending on the disease severity. This finding raises the possibility of using probiotics to manage brain inflammation in MS (136). A study shows that supplementation of *Prevotella histicola* suppressed autoimmune encephalomyelitis (EAE) in the HLA-DR3.DQ8 transgenic mouse model (137). This model expresses HLA-DR3 and DQ8 genes and can develop EAE, a severe spinal cord and brain inflammation that is very comparable to MS in human (138). In a follow-up study, the authors found that treatment with *P. histicola* yielded a similar disease-suppression effect as the MS drug Copaxone. However, co-administration of both *P. histicola* and Copaxone does not provide a synergic effect. Copaxone acts by decreasing the response of antigenic T cells in the brain (139). Data show that treatment with *P. histicola* increased the level of regulatory T cells and decreased proinflammatory cells, particularly those producing IL-17 and IFN-γ (140). Interestingly, a study shows that microbial transplant from MS patients developed EAE in transgenic mice (141). These results are a promising development to microbiome-based therapeutics for autoimmune diseases. However, some preclinical data claim that the beneficial effects of probiotics in delaying MS progression (142) are likely through indirect anti-inflammatory and immune-modulatory activity.

A recent study suggests a strong link between lung microbiome dysbiosis and MS in rats. Microbiome shift to a less lipopolysaccharide-producing phyla escalates MS while its enrichment decreases the proinflammatory response (143). The mechanistic underpinning is impairment in the responsiveness of microglial cells in the brain to type II interferons, resulting in a reduced recruitment of immune cells and further clinical manifestations (143).

Activation of latent Epstein–Barr virus (EBV) infection is linked to the development of MS (144, 145). EBV is a common virus that is considered part of the commensal microbiome (146). EBV infects B cells and epithelial cells, and because it shares molecular mimicry to some host protein, the viral genome integrates within the host DNA. When triggered, by yet unknown signals, it can lead to systemic autoimmune diseases. A recent study shows that EBV antibodies are associated with 99% of MS cases with the US military (145). The authors identified a strong positive association between MS and EBV where EBV infection increases the risk of developing MS by 32% (145) and MS only develops after EBV infection. If EBV is truly a prerequisite to MS, this discovery holds the promise of turning these untreatable diseases into vaccine-preventable ones.

### The role of microbial dysbiosis in triggering rheumatoid arthritis

RA is a systemic autoimmune disease affecting joints, and sometimes other internal organs, causing inflammation and swelling. One of the first reports of the connection between microbial dysbiosis and RA dates back to 1979 with the discovery that germ-free rats are 100% susceptible to developing RA upon injection of an intradermal adjuvant (147), while conventional rats are only 0 to 20% susceptible and further develop weak or delayed inflammation (147). Interestingly, this induced inflammation is resolved by inoculation of *E. coli* and slightly worsens by inoculation of *Lactobacilli* (148). The authors claimed a possible role of the LPS of *E. coli* in resolving RA (138). Studies show that the microbiome in RA patients is enriched in specific microbial taxa such as *Provetella*, *Lactobacillus sabotage*, and segmented filamentous bacteria (62). Higher abundances of these microbes increase their proinflammatory metabolites such as serum amyloid protein A, which increases the production of Th1 and Th17 that migrate systemically and contribute to the diseases (62). Some members of the *Provetella* genus are implicated in several inflammatory and autoimmune conditions that range from low-grade inflammation to periodontitis, bacterial vaginosis, and RA (67).

**Figure 4:**
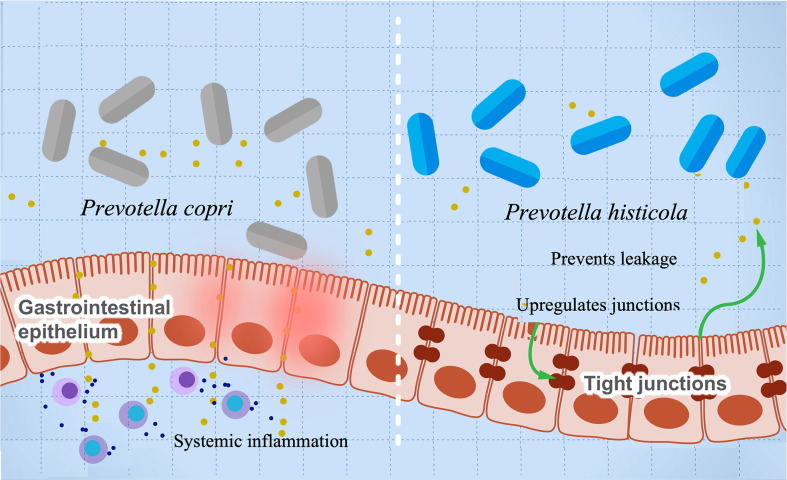
The influence of *Prevotella* species in preventing or mediating RA. The figure illustrates the role of *P. histicola* in upregulating the tight junctions, which prevent leakage of proinflammatory metabolites and subsequently prevents inflammation (right side). On the contrary, *P. copri* stimulates differentiation of Th17, leading to upregulation of proinflammatory cytokines’ production and systemic inflammation.

A growing body of research suggests a possible role of *Provetella copri* in the development of RA (63–67). Mechanistically, the ability of *Prevotella copri* to induce mucosal inflammation is due to the activation of TLR-2 stimulating the differentiation of Th17 and leading to the excessive production of proinflammatory interleukins such as IL-23 and IL-1 and the recruitment of neutrophils (67, 149). This inflammation increases gut permeability and leakage of microbes and proinflammatory molecules, leading to more systemic inflammation and immune reaction. A higher abundance of *P. copri* is also associated with RA in mice with knocked out *NLRP6* gene, a proinflammatory gene that is part of the inflammasome (63). These mice are genetically modified to produce lower levels of proinflammatory cytokines. Co-housing of wild-type and knockout mice resulted in inflammatory symptoms in the wild type and suggests that gut microbes can induce autoimmune diseases even without a genetic predisposition. This finding also suggests a possible role of *Provetella* in arthritis etiology as a pathobiont, and it could be potentially used as a diagnostic biomarker for RA (150). *Prevotella intestinalis* is another member of the family implicated in intestinal inflammation and particularly colitis through a reduction in SCFAs and the anti-inflammatory interleukin IL-18 in mice (68). Other studies show that *P. histicola* has anti-inflammatory activity and can protect mice from arthritis (55). Interestingly, the mechanism of inflammation suppression looks opposite to that induced by other pathobionts from the same genus. The authors show that *P. histicola* regulates DCs (CD103+), resulting in the generation of Treg cells, which suppresses Th17, decreasing proinflammatory interleukins, and increasing anti-inflammatory interleukins such as IL-10 (55). In addition, *P. histicola* upregulates the production of the tight junction protein, which decreases gut permeability (55). These findings suggest a potential application of *P. histicola* as a probiotic for arthritis and possibly other autoimmune diseases (151). **Figure 4** illustrates the paradoxical activities of *P. copri* and *P. histicola* in mediating RA.

### The role of microbial dysbiosis in triggering systemic lupus erythematosus

SLE is an autoimmune disease that affects joints, blood, kidney, and other organs with yet an elusive etiology. The hallmark of SLE is the formation and deposition of immune complexes from the production of autoantibodies directed towards nuclear antigen and could be detected several years before the onset of the disease (152–154). This autoimmune attack results in inflammation and organ failure (155). The mechanism beyond stimulation of autoreactive T cells and autoantibody production is still unclear, but various theories exist such as genetic deposition or environmental factors exhibiting molecular mimicry (153–155). This inflammation is increasingly believed to be attributed to an imbalance in Th17 and Treg cells, leading to a higher production of proinflammatory cytokines such as IL-17, IL-22, and IL-23 (156, 157) that drive systemic inflammation. Evidently, a high level of IL-17-producing cells is reported to infiltrate tissues of kidney, lung, and skin of SLE patients, resulting in organ damage (158). Understanding the role of the human microbiota in driving, intensifying, or preventing SLE is gaining intriguing interest (159). A unique gut microbiota signature is reported with the inflammatory flares and as the disease progresses (160, 161). This signature is characterized by a reduction in microbiota diversity with enrichment in some genera such as *Campylobacter*, *Streptococcus*, and *Veillonella*, and depletion of others such as *Bifidobacterium* (159). A significantly low abundance of *Lactobacillus* and an increase in Lachnospiraceae are associated with SLE in mice (162). The enrichment in Lachnospiraceae is gender-specific, with more abundance in females, which increases the disease’s severity. Interestingly, lupus-susceptible mice showed enrichment in the metabolic pathways of motility and sporulation genes (162), which might be linked to the ability of microbes to cause systemic inflammation. Restoration of lactobacilli abundance by feeding retinoic acid resulted in improved symptoms of lupus, suggesting a potential role of lactobacilli in preventing or counteracting inflammation. Studies reported a shift in Firmicutes/Bacteroidetes ratio accompanied by a change in SCFAs and Th17 levels in serum of SEL patients (56, 163). The shifted microbiota in SEL is characterized by an imbalance in the Treg/Th17/Th1 ratio. Specifically, two strains from the genus *Clostridium* drive an imbalance in Th17/Th1, promoting differentiation of CD4+ lymphocytes into Th17 and resulting in inflammation (56). *Bifidobacterium bifidum* inhibits the excessive stimulation of CD4+ lymphocytes (56). Other studies show that microbiota translocation might be implemented in triggering autoimmune diseases including SLE (69, 164). A study showed that translocation of *Enterococcus gallinarum* from the gut to the liver resulted in overproduction of autoimmune antibodies, inflammation, and mortality in genetically susceptible mice (69). While antibiotic treatment aimed to eradicate *E. gallinarum*, it also eliminated the autoantibodies in mice. Interestingly DNA of *E. gallinarum* recovered from the liver of autoimmune patients induced proinflammation in human hepatocytes mimicking the interaction in mice (69). Activation of latent EBV infection is also linked to the development of SLE (165–167). The tumorigenic activity of EBV might resonate with its ability to evade the immune system. Antigens of EBV share molecular mimicry to SLE antigens, which leads to an autoimmune response during EBV activation (168, 169). Furthermore, EBV suppresses the anti-inflammatory interleukins, resulting in more systemic inflammation (168, 169). A trial EBV peptide vaccine in experimental animals generated cross-reactive antibodies and caused SLE-like symptoms (170, 171). Although EBV is known to induce a transit increase in autoantibodies and inflammation, some studies show that this inflammation can further escalate and spread systemically (172, 173).

## Microbiome-based therapeutics for tackling autoimmune diseases

The prevalence of autoimmune diseases and allergies especially in children increased 40% over the last decade (174), resulting from the change in lifestyles such as diet, stress, and pollution. These changes result in a significant shift in microbiome composition (175). A recent study reported a strain-level significant microbiome signature in pediatric allergy characterized by a higher abundance of *Ruminococcus gnavus*, which is enriched in genes for the production of proinflammatory polysaccharides and lowers the potential for fiber-degrading enzymes (76). An interesting study shows that gut microbes drive sex-biased regulation of autoimmunity by directly regulating serum testosterone in NOD mice (176, 177). The authors show that an elevated testosterone level protects against T1D in male mice, and this protection is transferable to immature female mice by microbiome transplant (177). Another interesting study suggests that gut fungi induce behavioral change in mice through stimulation of immune response mediated by IL17, which binds to receptors in the brain (178).

### Manipulating the virome to control autoimmune diseases

One of the striking findings is the ability of some gut viruses of the *Iridoviridae* family to produce insulin-like molecules that mimic host insulin by 50% and can form the critical 3D molecules needed to bind and activate the insulin receptors (179). Interestingly, these viruses are primarily sequenced from fish (180) and recently have been identified in human fecal genomes (181). Recent studies show that the presence of LCDV-Sa is a risk factor for developing T1D in children (182). However, it is unclear if the presence of these insulin-mimic molecules helps to trigger diabetes or protect from diabetes. Previous studies show that microbial metabolites that mimic the host-derived molecules can trigger an immune reaction against insulin-producing cells resulting in T1D. Although we know much about the structural diversity of the microbiome bacteria, relatively much less research has been done on the human virome. A recent study identified 1,700 viral species in the gut microbiome. However, to date, only 2% of viruses are sequenced. This makes the virome research and its association with human diseases a very exciting area of development that will certainly advance our understanding of the microbe’s host interaction and association with human diseases. The use of phages to modulate the microbiome is still an unexplored avenue with unpredicted interspecies interactions (114).

### Tackling autoimmune diseases by microbial transplant or microbial metabolites

A study shows that germ-free mice remain immune-compromised even if they are colonized with animal or human microbiota (183). An interesting approach is the fecal transplant of gut microbes from a healthy donor to patients with autoimmune diseases. A randomized controlled clinical trial shows that fecal microbial transplant in newly diagnosed T1D patients prevents the further decline of insulin production by stabilizing the pancreatic B cells’ function (50). This effect is associated with a shift in plasma microbial metabolites, autoreactive T cells, and gene expression of the intestine (50). This trial not only provides hope for microbiome-based interventions for the treatment of autoimmune diseases but also provides solid evidence that microbiota dysbiosis drives T1D. Another possible scenario is the use of critical microbial metabolites such as butyrate as a supplement. Since butyrate has a protective role against autoimmunity (97), a diet rich in butyrate might dial down the autoimmune reaction. A study in NOD mice shows that butyrate and acetate supplementation decreased inflammation and might be a good candidate for therapeutic interventions to control autoimmune diseases such as T1D.

## Conclusion

Each living organism requires specific microbial species that are coevolved to prime the immune system (183). Changing the microbiome structure impairs important functions such as (1) gut permeability leading to leakage of antigens and inflammatory mediators to the blood circulation, (2) inability to produce anti-inflammatory microbial metabolites or to degrade food, and (3) loss of immune homeostasis leading to allergy and autoimmune reactions (6, 184–186). Understanding how gut microbes drive or suppress autoimmune diseases is crucial to developing innovative microbiome-based diagnostic tools and therapeutics.

## Author contributions

SH and FC collected data on multiple sclerosis. WM curated all data; reviewed the literature; critically analyzed the current knowledge, gaps, challenges, and future directions; designed/developed tables and figures; and wrote and edited the manuscript. All authors contributed to the article and approved the submitted version.

## Funding

This work is supported by a start-up fund from Whitman College and the Murdock Foundation.

## Conflict of interest

The authors declare that the research was conducted in the absence of any commercial or financial relationships that could be construed as a potential conflict of interest.

## Publisher’s note

All claims expressed in this article are solely those of the authors and do not necessarily represent those of their affiliated organizations, or those of the publisher, the editors and the reviewers. Any product that may be evaluated in this article, or claim that may be made by its manufacturer, is not guaranteed or endorsed by the publisher.
